# Genome-Wide Identification of Na^+^/H^+^ Antiporter (NHX) Genes in Sugar Beet (*Beta vulgaris* L.) and Their Regulated Expression under Salt Stress

**DOI:** 10.3390/genes10050401

**Published:** 2019-05-27

**Authors:** Guo-Qiang Wu, Jin-Long Wang, Shan-Jia Li

**Affiliations:** School of Life Science and Engineering, Lanzhou University of Technology, Lanzhou 730050, China; jinlongwang0112@163.com (J.-L.W.); lishanjia@lut.cn (S.-J.L.)

**Keywords:** sugar beet, Na^+^/H^+^ antiporter, amiloride-binding site, Na^+^ compartmentalization, Na^+^ exclusion, salt tolerance

## Abstract

Salinity is one of the major environment factors that limits the growth of plants and the productivity of crops worldwide. It has been shown that Na^+^ transporters play a central role in salt tolerance and development of plants. The objective of this study was to identify Na^+^/H^+^ antiporter (NHX) genes and investigate their expression patterns in sugar beet (*Beta vulgaris* L.) subjected to various concentrations of NaCl. A total of five putative *NHX* genes were identified and distributed on four chromosomes in sugar beet. Phylogenetic analysis revealed that these *BvNHX* genes are grouped into three major classes, viz Vac- (*BvNHX1*, *-2* and *-3*), Endo- (*BvNHX4*), and PM-class *NHX* (*BvNHX5/BvSOS1*), and within each class the exon/intron structures are conserved. The amiloride-binding site is found in TM3 at N-terminus of Vac-class NHX proteins. Protein-protein interaction (PPI) prediction suggested that only BvNHX5 putatively interacts with calcineurin B-like proteins (CBL) and CBL-interacting protein kinases (CIPK), implying it might be the primary NHX involved in CBL-CIPK pathway under saline condition. It was also found that *BvNHX5* contains one abscisic acid (ABA)-responsive element (ABRE), suggesting that *BvNHX5* might be involved in ABA signal responsiveness. Additionally, the qRT-PCR analysis showed that all the *BvNHX* genes in both roots and leaves are significantly up-regulated by salt, and the transcription levels under high salinity are significantly higher than those under either low or moderate salinity. Taken together, this work gives a detailed overview of the *BvNHX* genes and their expression patterns under salt stress. Our findings also provide useful information for elucidating the molecular mechanisms of Na^+^ homeostasis and further functional identification of the *BvNHX* genes in sugar beet.

## 1. Introduction

Salinity is one of the major environment factors that limits crops productivity worldwide [1]. It is estimated that approximately 20% of cultivated land and one half of irrigated land worldwide suffers salinity damage [2]. Salt stress has a vital effect on the growth and development of plants [3]. High saline soils reduce the ability to uptake water and nutrients, resulting in osmotic or water-deficit stress [4]. To cope with salt stress, plants have evolved a series of smart and precise mechanisms, including regulation of growth and development, ion homeostasis, detoxification, and osmotic adjustment [5]. Of these, the maintenance of ion homeostasis is one of the most important strategies for plants adaptive to salt stress [6].

It is well-known that Na^+^/H^+^ antiporters (NHXs), which are located on plasma membranes and tonoplast, play a central role in the maintenance of Na^+^ homeostasis by transporting Na^+^ from the cytoplasm into either the extracellular spaces or vacuoles [7]. They are driven by the H^+^ electrochemical gradient generated by two different kinds of proton pumps, viz H^+^-ATPase and H^+^-PPase [8,9]. In *Arabidopsis thaliana*, the *NHX* gene families have eight members that are divided into three major classes based on their subcellular localization [10]. *AtNHX1, -2, -3*, and *-4* are located on vacuolar membranes and named Vac-class *NHXs* [11], *AtNHX5* and *AtNHX6* are located on the endosomal compartment and named Endo-class *NHXs* [12], while *AtNHX7* (also named *AtSOS1*) and *AtNHX8* are located on plasma membranes and named PM-class [13]. Most NHX proteins have been shown to contain 10–12 transmembrane helix domains (TMs), and the amiloride-binding site (FFIYLLPPI), a typical feature of Vac-class NHX proteins, is found in TM3 at N-terminus [14].

It is well documented that the NHX proteins are involved in cell expansion [15], pH regulation [16], salt stress response [17], K^+^ homeostasis [18,19], long-distance Na^+^ transport [20], and cellular vesicle trafficking [21]. Overexpression of *Reaumuria trigyna RtNHX1* leads to accumulate more K^+^ and less Na^+^ in transgenic *Arabidopsis* compared to wild-type (WT) plants [22]. The ectopic expression of *Zygophyllum xanthoxylum ZxNHX*, together with *ZxVP1-1*, a H^+^-PPase gene in alfalfa (*Medicago sativa*), confers plant tolerances to both salt stress and water-deficit stress [23]. These results suggested that the *NHX* genes have important application values in the improvement of stress tolerance in crops.

Sugar beet (*Beta vulgaris* L.), which belongs to Chenopodiaceae family, is one of the most important sugar crops worldwide [24] and provides approximately 30% of the world’s annual sugar production [25]. In China, it is also the second largest sugar crop and cultivated mainly in the arid and semi-arid areas of Northern China, where irrigation is the most effective method to maintain a high yield of crops [26]. Sugar beet was used as an important source not only for animal feed but also for bioethanol production [27,28]. Previously, Wu et al. [26] compared salt tolerance among three sugar beet cultivars by combining physiological and agronomic criteria, and found that cultivar “Gantang7” is more tolerant to salt stress than the other two cultivars. Further studies showed that an additional 50 mM NaCl stimulates the growth of plants and enhances the tolerance to osmotic stress in sugar beet [29]. Sugar beet is thought to be a salt tolerant crop [26,29], compared to other species, such as *Arabidopsis*, wheat, and alfalfa. Recently, the genome sequence of sugar beet has been completed [30], and this makes it possible to identify the *NHX* genes at the whole genome level. Previous studies have shown that *BvNHX1* might play a key role in sugar beet response to salt stress [31]. However, the comprehensive information and functional characterization of the *NHX* gene families of sugar beet still remain unknown.

Here, we proposed a hypothesis that the *NHX* genes might be involved in response to salt in sugar beet. To test this hypothesis, firstly, a total of five *NHX* genes were identified in the sugar beet genome, and their structures, phylogenetic relationship, chromosomal localizations, putative protein-interaction-protein (PPI) network, conserved motifs, and three-dimensional (3-D) structures were systematically analyzed; secondly, their regulated expression patterns under salt stress were investigated. Our findings shed light on the molecular properties and evolutionary relationship of the *BvNHX* family and provide useful theoretical support for future in-depth elucidation of biological functions of the *NHX* genes under salt stress. 

## 2. Materials and Methods

### 2.1. Identification and Characterization of the *NHX* Genes in Sugar Beet

The sequences of 8 *Arabidopsis AtNHXs* were obtained from the TAIR database (https://www.arabidopsis.org/) [32] and then used search *BvNHXs* with the BLASTP tool using the NCBI sugar beet genome (https://www.ncbi.nlm.nih.gov/genome/?term=Beta+vulgaris) and genome database of sugar beet (http://bvseq.boku.ac.at/index.shtml) [30]. All homologous protein sequences of the *NHX* candidates are accepted if they are satisfied with the expectation value (*E*) < 10^−40^ [33].

The isoelectric point (pI) and molecular weight (MW) of *BvNHXs* were computed by ExPASy (https://web.expasy.org/compute_pi/) [34]. The subcellular localization of *BvNHX* was predicted with the Plant-mPLoc server (http://www.csbio.sjtu.edu.cn/bioinf/plant-multi/) [35].

To further confirm the transmembrane helical domains (TMs) in NHXs, the candidate sequences were scanned with TMHMM server version 2.0 (http://www.cbs.dtu.dk/services/TMHMM/). Phosphorylation sites of *BvNHX* are predicted by NetPhos 3.1 server (http://www.cbs.dtu.dk/services/NetPhos/) [36].

### 2.2. Phylogenetic Analysis

To investigate the phylogenetic relationship between BvNHXs and other NHXs from various plants species, BvNHXs were aligned NHXs from *Arabidopsis thaliana* (*At*), *Cucurbita maxima* (*Cm*), *Eutrema halophilum* (*Eh*), *Hordeum vulgare* (*Hv*), *Gossypium hirsutum* (*Gh*), *Oryza sativa* (*Os*), *Solanum lycopersicum* (*Sl*), *Solanum tuberosum* (*St*), *Sorghum bicolor* (*Sb*), *Spinacia oleracea* (*So*), *Triticum aestivum* (*Ta*), and *Vitis vinifera* (*Vv*), by using Clustal W version 2.1 software. All the sequences and accession number of *NHX* genes are listed in Supplementary Table S1. Phylogenetic tree was constructed by MEGA7.0 (https://www.megasoftware.net/history.php) using the neighbor-joining (NJ) method, with 1000 bootstrap replicates [37].

### 2.3. Chromosome Distributioof *BvNHX* Genes and Analysis of Ka/Ks Ratio

The physical positions of the *BvNHX* genes along each chromosome were identified from the sugar beet genome database and the distribution graph of *BvNHX* genes was drawn by MapInspect 1.0 software (https://mapinspect.software.informer.com/). Rate of synonymous (*Ks*) and non-synonymous (*Ka*) substitution were estimated using the PAL2NAL program (http://www.bork.embl.de/pal2nal/) [38].

### 2.4. Analysis of Conserved Motifs, Gene Structures, and cis-Acting Elements 

The conserved motifs in the NHX proteins were identified with Multiple Expectation Maximization for Motif Elicitation program (MEME version 5.0.5, http://meme-suite.org/tools/meme) with the following parameter settings: The maximum number of motifs is 16, and the optimum width is set from 6–50. 

To analyze gene structure, the exon/intron of the *BvNHX* genes were generated using Gene Structure Display Serve (GSDS, http://gsds.cbi.pku.edu.cn/) [39] by aligning the CDS sequences with the corresponding genomic DNA sequences from the genome database of sugar beet (http://bvseq.boku.ac.at/index.shtml) [30].

To identify the various *cis*-acting regulatory elements in promoters of the *BvNHX* genes, 1500 bp upstream of the CDS was estimated using PlantCARE software (http://bioinformatics.psb.ugent.be/webtools/plantcare/html/) [40].

### 2.5. Three-Dimensional Structural Prediction of BvNHX Proteins

The three-dimensional (3-D) structure of BvNHX proteins were predicted using the I-TASSER program (https://zhanglab.ccmb.med.umich.edu/I-TASSER/) [41]. To identify the best structural template for BvNHX in the Protein Data Bank (PDB) database [42], the query sequences were subjected to multiple rounds of threading using LOMETS [43].

### 2.6. Protein-Protein Interaction Prediction of BvNHX Proteins

The protein-protein interaction (PPI) of BvNHX proteins was predicted by STRING database (http://string-db.org) [44].

### 2.7. Plant Material, Treatment, and qRT-PCR Analysis

Seeds of sugar beet (*B. vulgaris* L.) cultivar “Gantang7”, which is a salt-tolerant cultivar [26], were sterilized for 3 min with 75% ethanol (*v*/*v*) and rinsed three times with distilled water, soaked in distilled water overnight, and then germinated at 25 °C in the dark for three days. Uniform seedlings were transferred to a plugged hole in plastic containers (5 cm × 5 cm × 5 cm; 2 seedlings/container) filled with the distilled vermiculite and irrigated with modified Hoagland nutrient solution (2.5 mM KNO_3_, 1 mM NH_4_H_2_PO_4_, 0.5 mM MgSO_4_, 0.5 mM Ca(NO_3_)_2_, 0.7 µM (NH_4_)_6_Mo_7_O_24_·4H_2_O, 60 µM Fe-Citrate, 92 µM H_3_BO_3_, 1.6 µM ZnSO_4_·7H_2_O, 18 µM MnCl_2_·4H_2_O, and 0.6 µM CuSO_4_·5H_2_O). The seedlings were grown in the same chamber with the temperature of 20/25 °C (night/day), the daily photoperiod of 8/16 h (night/day), the light flux density of 550–600 µmol·m^−2^·s^−1^, and the relative humidity of 65–75%. Solutions were renewed every three days.

Four-week-old seedlings were treated with modified Hoagland nutrient solution supplemented with 50, 100, 200, and 300 mM NaCl [45], and the plants were harvested at 0, 3, 6, 12, 24, and 48 h after treatments of salt, respectively. The roots of plants were washed three times with distilled water to remove vermiculite and the leaves were rinsed in deionized water to remove surface salts. The whole leaves and roots were separated and harvested, in three biological replicates for RAN preparation. There are two plants in each replicate. All the harvested samples were immediately frozen in liquid nitrogen and stored at −80 °C until use. 

Total RNA was isolated from each sample using a Trizol Total RNA Isolation Kit (Sangon, Shanghai, China). cDNA synthesis was carried out using a PrimeScript™ RT Master Mix Kit (Takara, Dalian, China). All the sequences of primers used for qRT-PCR are shown in Table 1. *BvACTIN* was used as a constitute expression control in qRT-PCR experiments. qRT-PCR was performed using a MA-6000 Real-Time PCR System (Molarray, Suzhou, China) with a TB Green^TM^ Premix Ex Taq^TM^ II Kit (Takara, Dalian, China). The conditions followed for the experiment are 95 °C for 30 s, and 40 cycles of 95 °C for 5 s and 60 °C for 60 s. Three biological repeats are used at least. The relative expression levels of the *BvNHX* genes are represented in the form of relative changes by the 2^−ΔΔCt^ method [46].

## 3. Results

### 3.1. Identification of *BvNHX* Genes

To investigate the *BvNHX* gene families in sugar beet, the peptides of *AtNHX* of *Arabidopsis* were used as queries to screen the sugar beet genome in silico (Supplementary Table S1). The results showed that a total of five full-length genes coding putative Na^+^/H^+^ antiporter (NHX) was identified in the sugar beet genome and the sequences were downloaded from the sugar beet genome database (Supplementary Table Sl). All the *NHX* genes are assigned specific names (Table 2). The sequence analysis of these *BvNHX* genes showed that CDS ranges from 1560 bp (*BvNHX2*) to 3489 bp (*BvNHX5*) and the predicted protein varies from 519–1162 amino acids in length. Additionally, the molecular weights (MW) of the BvNHX proteins range from 58.2–128.5 and the isoelectric points (pI) range from 5.5–8.45 (Table 2). The number of transmembrane helical domains (TMs) in NHX proteins varies from 11–12 (Table 2 and Supplementary Figure S1). 

Subcellular localization analysis indicated that three genes, *BvNHX1*, *-2*, and *-3*, are localized on the vacuole (Vac), *BvNHX4* is localized on the endosome (Endo), while *BvNHX5*, also named *BvSOS1*, is localized on the plasma membrane (PM). BvNHX phosphorylation sites vary in number, for serine 20 (BvNHX2) to 64 (BvNHX5), threonine 11 (BvNHX4) to 36 (BvNHX5), while tyrosine range from 2 (BvNHX2 and -4) to 8 (BvNHX5) (Supplementary Table S1), indicating serine is the most common site for phosphorylation with comparison to tyrosine and threonine. Additionally, BvNHX proteins have been found to be more phosphorylated with protein kinase A (PKA), protein kinase C (PKC), and cell division cycle protein 2 (CDC2), and less with ataxia telangiectasia mutated (ATM) and glycogen synthase kinase 3 (GSK3) (Supplementary Table S1).

### 3.2. Phylogenetic Relationship of Sugar Beet and Other Plants in NHX Gene Families

**Figure 1:**
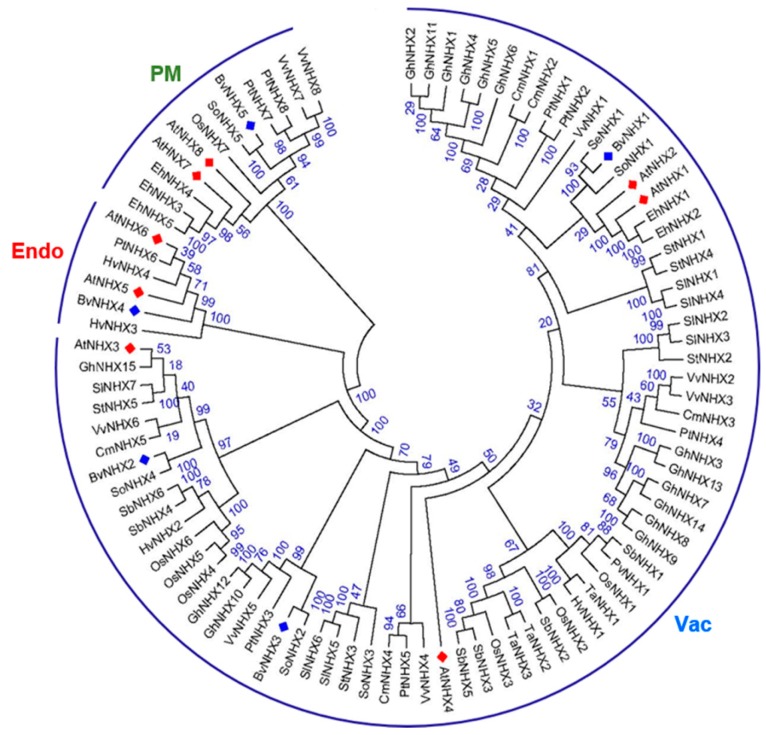
Phylogenetic tree of the NHX genes from Beta vulgaris (Bv), Arabidopsis thaliana (At), Cucurbita maxima (Cm), Eutrema halophilum (Eh), Hordeum vulgare (Hv), Gossypium hirsutum (Gh), O. sativa (Os), Solanum lycopersicum (Sl), Sorghum bicolor (Sb), Spinacia oleracea (So), Solanum tuberosum (St), Triticum aestivum (Ta) and Vitis vinifera (Vv). The tree was determined by the Neighbor-Joining method (NJ) with 1000 bootstrap replicates using MEGA7.0. According to the clustering of the NHX proteins, we divided proteins into three different classes, viz Vac-, Endo-, and PM-class. Proteins from sugar beet and Arabidopsis are denoted by blue and red diamonds, respectively. Details of NHXs from 13 species are listed in Supplementary Table S1.

To determine the evolutionary relationship of the *NHX* gene families in higher plants, *BvNHXs* with *NHXs* from other 12 species are compared. Of these, eight dicotyledonous angiosperms: *A. thaliana* (*At*), *C. maxima* (*Cm*), *E. halophilum* (*Eh*), *G. hirsutum* (*Gh*), *S. oleracea* (*So*), *S. tuberosum* (*St*), *S. lycopersicum* (*Sl*), and *V. vinifera* (*Vv*); four monocotyledonous angiosperms: *H. vulgare* (*Hv*), *O. sativa* (*Os*), *S. bicolor* (*Sb*) and *T. aestivum* (*Ta*) were analyzed. Then, a phylogenetic tree was built using 93 genes from 13 plant species using MEGA7.0 software. Protein sequence alignment showed that *NHX* genes are clustered into three subfamilies, designated Vac-class, Endo-class, and PM-class (Figure 1). With Vac-class *NHXs* the most abundant from all the investigated species.

### 3.3. Chromosomal Location, Ka/Ks Ratio Calculation and Gene Structure Analysis of *BvNHX* Genes

**Figure 2:**
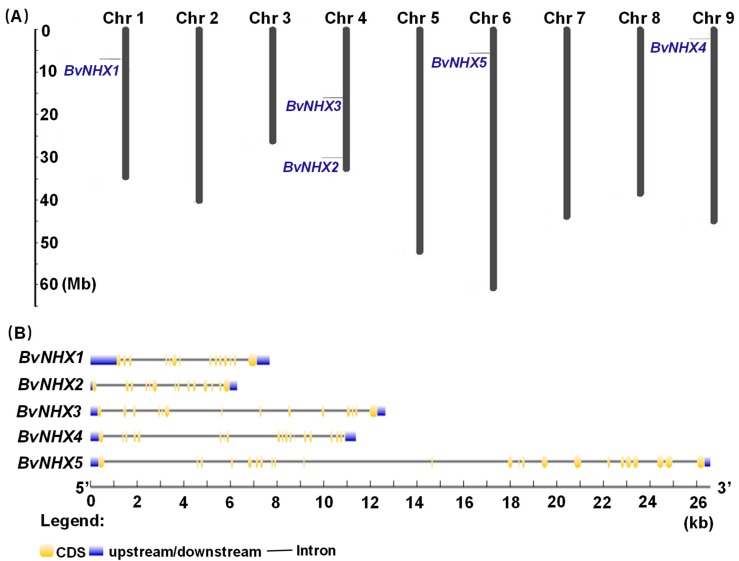
Physical mapping and structure analysis of the *BvNHX* gene. (**A**) The distribution of the *BvNHX* genes of sugar beet on 9 chromosomes. The number of chromosomes is shown at the top of each chromosome. The numbers of *BvNHX* genes are indicated on the left of each chromosome. The scale of the genome size is given on the left. (**B**) Gene structures of the *BvNHX* genes. Exons are indicated by yellow boxes. Introns are indicated by black lines. Upstream and downstream are indicated by blue boxes. The scale of genes length is given at the bottom. CDS: Coding sequence; Mb: million bases; kb: kilo bases.

In order to examine the genome distribution of the *BvNHX* genes, chromosomal mapping was performed by MapInspect 1.0 software. As shown in Figure 2A, five *BvNHX* genes are mapped onto four of total 9 sugar beet chromosomes, indicating a diverse distribution. Two genes (*BvNHX2* and *-3*) are located on chromosomal 4, while *BvNHX1*, *-4*, and *-5* are found on chromosomal 1, 9, and 6, respectively.

To further identify the structural characteristics of the *BvNHX* genes, the exon/intron organizations of these genes were analyzed and compared (Figure 2B). *BvNHX1–3* (Vac-class) have 13 introns, *BvNHX4* (Endo-class) contains 18 introns, whilst *BvNHX5* (PM-class) possesses 23 introns. The exon length, intron number, and intron phase are relatively conserved among members of Vac-class *NHX*. Additionally, the conservation of sequence among *BvNHX* genes was also confirmed by identities of amino acid sequences (Table 3). Two *BvNHX* paralogous pairs in Vac-class display higher identities of sequence in amino acid level (*BvNHX1*/*BvNHX2* = 78.8% and *BvNHX1*/*BvNHX3* = 79.6%), whilst the *BvNHX* genes in different subfamilies exhibit lower identities (8.6–10.1%). The sequences of *BvNHX1*/*AtNHX1* and *BvNHX5*/*AtNHX7* have higher identities (89.2% and 61.9%) (Table 3). Our data also showed that *BvNHX1* and *BvNHX2* display smaller divergence, while *BvNHX1* and *BvNHX5* have larger divergence (Table 3).

To investigate the selective pressure on *BvNHX* genes, the ratio of non-synonymous/synonymous (*Ka*/*Ks*) was calculated. A *Ka*/*Ks* ratio > 1 suggests positive selection, *Ka*/*Ks* ratio = 1 shows neutral selection, while ratio of *Ka*/*Ks* < 1 suggests purifying selection [47]. In the present study, *Ka*/*Ks* ratio between *BvNHX3* and *BvNHX5* has been found to be 0.1855 (Table 4), implying that the genes underwent a purifying selection or a positive Darwinian selection. 

### 3.4. Analysis of the Conserved Motifs of BvNHX Proteins

**Figure 3:**
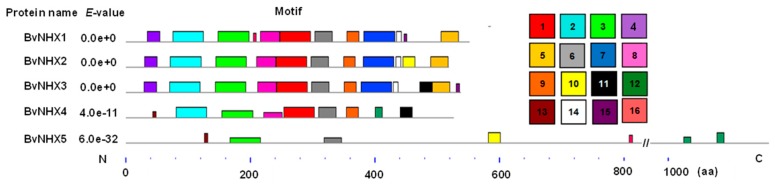
Motif analysis of the BvNHX proteins. Distribution of conserved motifs in BvNHXs was analyzed by the online tool MEME. Details of different motifs indicated by different colors are shown in Supplementary Figure S2. The scale of proteins length was given at the bottom. aa: amino acid.

To better understand the structural diversity of BvNHX proteins, the motif distributions in the proteins were investigated using the MEME program which identified a total of 16 putative motifs (Figure 3 and Supplementary Figure S2). The predicted motifs of BvNHX range from 6–50 amino acids in length. Motifs 3 and 6 are existed in all the members in BvNHX family and these motifs are located in N-terminus of BvNHX. Four motifs (motifs 1, 2, 8, and 9) are existed in both Vac- and Endo-class NHXs (BvNHX1, -2, -3 and -4), whilst other four motifs (motifs 4, 5, 7, and 14) are only detected in Vac-class NHXs (BvNHX1, -2 and -3). Motif 10 is existed in both BvNHX2 and -5. Motifs 12 and 13 are existed in BvNHX4 and -5 (Figure 3 and Supplementary Figure S2). Noticeably, amiloride-binding site (FFIYLLPPI), which is a typical feature of NHX proteins, is found in motif 2 of BvNHX1, -2 and -3, but absent in BvNHX4 and -5.

### 3.5. Analysis of cis-Acting Elements in *BvNHX* Promoters

To further explore the regulatory role of the *BvNHX* genes, the *cis*-acting elements of upstream region in *BvNHX* genes were predicted using the PlantCARE tool. As shown in Table 5, amounts of hormone-related (e.g., ABA, ethylene, salicylic acid (SA), and auxin), stress-related (e.g., anaerobic, drought, low temperature, wound, and salt) and development-related (e.g., zein metabolism regulation and light response) were identified in the *BvNHX* promoters. Among hormone-related *cis*-acting regulatory elements, ABRE (abscisic acid-responsive element) is found in *BvNHX5* promoter, while ERE (ethylene-responsive element) are found in the promoters of *BvNHX1*, *-3*, and *-4*. Among stress-related *cis*-acting elements, DRE (drought-responsive element) and MYB are found in the *BvNHX1* promoter, while LTR (low temperature-responsive element) and W box (salt-responsive element) are predicted in the *BvNHX4* promoter. These results implied that *BvNHXs* might have potential roles in hormone signal responsiveness and stress adaptation.

### 3.6. Analysis of BvNHX Proteins Structures

**Figure 4:**
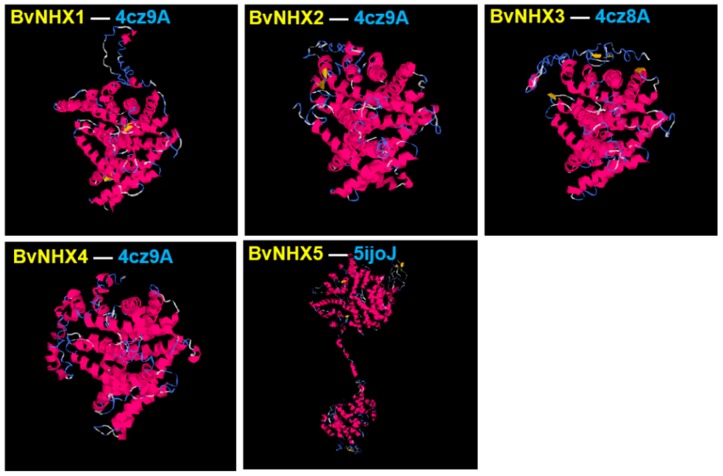
Structural analysis of five BvNHX modeled proteins. The best PDB structural analog for each transporter is shown in Table 6. Details of secondary structure of BvNHX proteins are shown in Supplementary Figure S3.

To understand the putatively functional mechanism of NHX proteins in sugar beet, all the BvNHX proteins are modeled by I-TASSER software. The 3-D structures were construed according to the similar structural templates and crystal structures obtained from PDB (Protein Data Bank) (Figure 4). C-score was used to estimate the confidence of the constructed protein model for each BvNHX protein [33]. C-score typically ranges from −5 to 2, a higher value represents a model with a higher confidence and vice versa. In the present study, all the predicted BvNHX models have a C-score range from −1.83 to −0.61 (Table 6), suggesting the structures of BvNHXs are constructed with high accuracy.

### 3.7. Protein-Protein Interaction Prediction of BvNHXs

**Figure 5:**
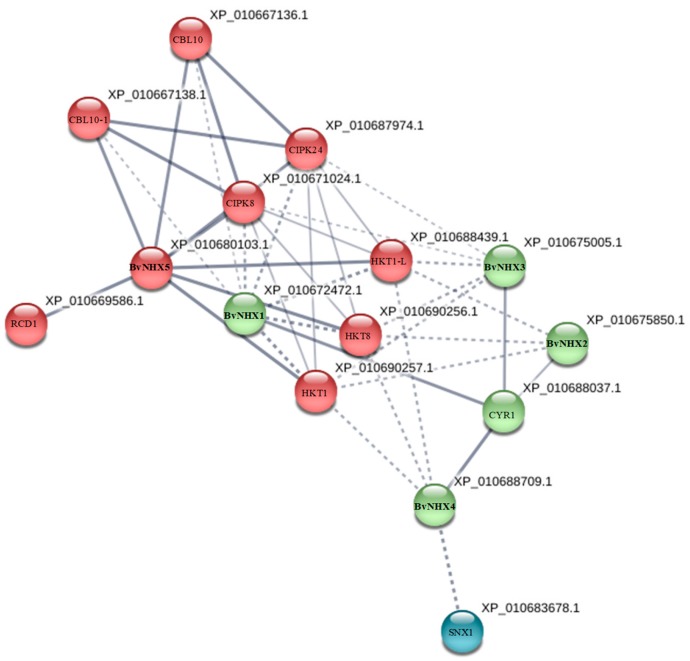
Protein-protein interaction (PPI) network of BvNHXs. Line thickness indicates the strength of data support. Network is clustered to 3 clusters, which are represented with red, green, and blue nodes, respectively. Details of string analysis for individual BvNHX protein are shown in Supplementary Figure S4.

To further explore the potential function of BvNHXs during the possible interaction with other proteins, the PPI network is constructed by STRING database (Figure 5). No immediately interacted relationship is predicted among BvNHX proteins. However, BvNHX1, -2, -3, and -4 share the same putatively interacted protein, NADH-cytochrome *b5* reductase 1 (CYR1, XP_010688037.1). BvNHX5 is predicted to interact with some proteins, including CBL10 (XP_010667136.1), CIPK8 (X_010671024.1), CIPK24 (XP_010687974.1), HKT1 (XP_010690257.1), HKT8 (XP_010690256.1), and RCD1 (XP_010669586.1) (Figure 5). Additionally, individual BvNHX protein is also hypothesized to interact with other putative proteins, such as calmodulin (XP_010671757.1), CML18 (XP_010671108.1), H^+^-PPase (XP_010668498.1), V-H^+^-ATPase (XP_010693105.1), CLC (XP_010684687.1), and KEA3 (XP_010691482.1) (Supplementary Figure S4).

### 3.8. Expression Patterns of BvNHX under Various Concentrations of NaCl

**Figure 6:**
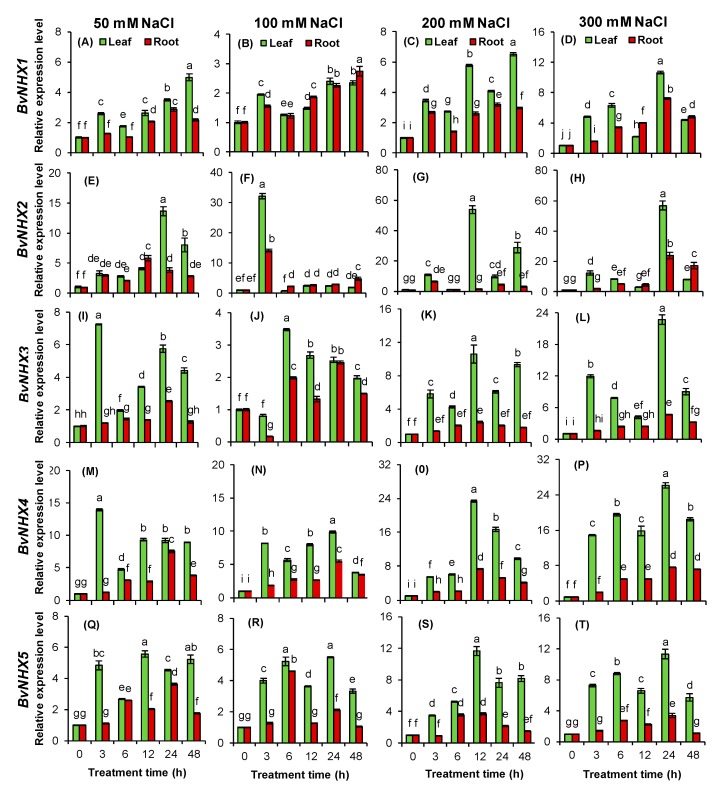
Relative expression levels of *BvNHX* genes in leaf and root of sugar beet seedlings subjected to 50, 100, 200, and 300 mM NaCl for 0, 3, 6, 12, 24, and 48 h. Expression of the *BvNHX* genes normalized to those of *BvACTIN* and shown relative to the expression at 0 h. The 2^−ΔΔCt^ method was used to calculate the expression levels of *BvNHX* genes at different time. Experiments were repeated at least 3 times. Values are means ± SE and bars indicate SE (*n = 3*). Columns with different letters represent significant differences at *p* < 0.05 level (Duncan’s test).

To further understand the possible functions of the *BvNHX* genes in response to salt stress, their expressional levels were investigated under different concentrations of NaCl over a 48-h period. The results showed that all the *BvNHX* genes are significantly induced by salt treatments (Figure 6). Interestingly, at low salt condition (50 mM NaCl), the expression of both *BvNHX1* and *BvNHX2* in leaf gradually increased over time until peak expression at 48 h and 24 h, respectively, which are 2.8- and 12.7-fold higher than those under control condition (0 h), while transcript of *BvNHX3*, *BvNHX4*, and *BvNHX5* are sharply up-regulated at short time (3 h) and then gradually increased. When exposed to moderate salt stress (100 mM NaCl), *BvNHX2* in leaf and root rapidly increased the expression level at 3 h, which was 31.2- and 14.1-fold higher than under control condition, respectively, and maintained a lower level from 6–48 h, while other *BvNHX* genes significantly increased their transcript abundances in either leaf or root over a 48-h period (except for *BvNHX3* at 3 h after treatment). Additionally, under high salt stresses (200 and 300 mM NaCl), it is worth noting that the mRNA levels of *BvNHX3*, *BvNHX4*, and *BvNHX5* in leaves are always significantly higher than those in roots at all treatment times.

## 4. Discussion

It is well-known that the plant *NHX* gene families encodes Na^+^/H^+^ antiporters which are crucial for ion homeostasis, cellular pH regulation, plant development, vesicle trafficking and salt tolerance [7,12,21]. In this study, the *NHX* genes were first identified from the sugar beet genome analysis and were further described by phylogenetic relationship, chromosomal localization, conserved motifs, 3-D structures, protein-protein interaction, and expression patterns under salt stress.

### 4.1. Identification and Structure Analysis of *BvNHX* Genes

In the present study, a total of five *BvNHX* genes have been identified in the genome of sugar beet (Table 1), like in other species such as tomato (*S. lycopersicum*) and potato (*S. tuberosum*), which also contain five *NHX* genes in all [18]. However, there are 10 *NHX* genes in soybean (*Glycine max*) [48], nine in maize (*Zea mays*) [18], eight in *Arabidopsis* [10] and poplar (*Populus trichocarpa*) [33], seven in *S. bicolor* [49] and rice (*O. sativa*) and [6], and six in *Chlamydomonas reinhardtii* [18]. These differences in the number of *NHX* genes in plants could be attributed to gene duplication and loss specific to different subfamilies of *NHX* over the course of evolution. 

Bioinformatics analysis showed that *NHX* members in sugar beet can be divided into three classes according to their vacuolar (*BvNHX1*, *BvNHX2,* and *BvNHX3*), endosomal (*BvNHX4*), and plasma membrane (*BvNHX5*/*BvSOS1*) localizations. It has been found that two endomembrane (*NHX5* and *NHX6*) and two plasma membrane antiporters (*NHX7* and *NHX8*) in *Arabidopsis* [10], only one is identified under these categories in sugar beet in this study (Figure 1). The exon/intron structural diversity, an important part in the evolution of gene families, provides additional evidence supporting phylogenetic groupings. In sugar beet, *BvNHX1*, *-2*, and *-3* have fewer exons (14–15) than *BvNHX4* (26) and *BvNHX5* (23) class members (Table 1 and Figure 2B). However, in poplar, Vac-class *NHXs* (*PtNHX1-5*) contain 14 exons, the Endo-class *NHX* (*PtNHX6*) has 22 exons, while the PM-class *NHXs* (*PtNHX7* and *PtNHX8*) display 23 exons [33]. Similarly, for *NHX* genes in soybean, seven members of *GmNHX* contain 14–15 exons, whereas other three members have 20 exons [48]. These results implied that there is structural diversification among the *NHX* genes families in plant species.

It has been shown that the putative amiloride-binding site and membrane-spanning pore are highly conserved in the *NHX* gene families [6,10,18], which consists of amino acid sequence “FFIYLLPPI” [18]. This domain serves to inhibit the cation/H^+^ exchange in the presence of drug amiloride and/or its derivatives [50]. In the present study, amiloride-binding site is located in TM3 of N-terminus, and it is found in BvNHX1, -2, and -3, but not in BvNHX4 and -5. Similar results are observed in AtNHX1-4 from *Arabidopsis* [11], IbNHX2 from sweetpotato (*Ipomoea batatas*) [50], and ZxNHX1 from *Z. xanthoxylum* [50]. Additionally, TM5 and -6 of BvNHX1, -3, and -4 are also highly conserved among NHX isoforms identified to date, which are considered critical for transport activity of antiporters [14]. Interestingly, these two regions do not appear to span the tonoplast membrane but yet appeared to be membrane-associated (Supplementary Figure S1). Similar domains are observed in AtNHX1 and ZxNHX1, which did not appear to be transmembrane segments [14,51].

### 4.2. Phylogenetic, Conserved Motif, and Promoter Analysis of *BvNHX* Genes

Previous studies demonstrated that *NHXs* in soybean, poplar, and *S. bicolor* were clustered in three groups [33,48,49]. Interestingly, members of *BvNHX* family were evolutionarily closer to those of *SoNHX* genes from spinach (*S. oleracea*), one member of Chenopodiaceae family, compared to *NHX* genes from other species (Figure 1). Additionally, conserved motif analysis showed that all the members of BvNHX contain motifs 3 and 6, whilst each subfamily of BvNHX shares the same conserved motifs (Figure 3 and Supplementary Figure S2). Similar results are found in PtNHXs [33] and SbNHXs [49]. These results indicated the *NHX* family genes are relatively conserved during the course of evolution. 

*Cis*-acting regulatory elements serve as key molecular switches involved in transcriptional regulation of the gene activities controlling various biological processes such as hormone response, abiotic stress response and development processes [52,53]. In plant, hormones, such as ABA, ethylene, SA, and IAA, play critical roles in a number of developmental stages and stress response [54,55,56,57]. In this study, *cis*-acting regulatory elements related to hormones are identified in the promoters of *BvNHX* genes (Table 5). *BvNHX5* has been found to contain one ABA responsive element (ABRE). Similar results were observed in poplar, where *PtNHX1–7* had one or two ABREs [33]. These results suggested that the *NHX* genes might be involved in ABA signal pathway. Furthermore, several stress responsive regulatory elements were identified namely ARE (involved in anaerobic induction, one in *BvNHX1* and two in *BvNHX4*), DRE (drought responsive *cis*-acting element, one in *BvNHX1*), LTR (low-temperature responsive element, one in *BvNHX4*), WUN-motif (wound-responsive element, one in *BvNHX3*), MYB (involved in drought response, one in *BvNHX1*), and STRE (involved in stress response, one in *BvNHX5*) (Table 5). Similar elements are found in *PeNHXs* from poplar and *SbNHXs* from *S. bicolor* [49]. Surprisingly, W-box, a DNA *cis*-acting regulatory element has also been detected in *BvNHX3*. W-box is recognized by the family of WRKY transcription factors which is involved in development processes and salt response in *Arabidopsis* [58]. Overall, the identified regulatory elements in the present study help in understanding their roles in the various abiotic and biotic stress related mechanisms. 

### 4.3. Expression Analysis of *BvNHX* Genes under Salt Stress

Sequestering Na^+^ into vacuoles is one of important strategies for plants to alleviate Na^+^ toxicity in cytoplasm under salinity stress [59]. The tonoplast Na^+^/H^+^ antiporters play crucial roles in sequestering Na^+^ into vacuoles to maintain Na^+^ homeostasis and, thus, to improve plant salinity tolerance [22]. In this study, the expression levels of both *BvNHX1* and *BvNHX3* are significantly up-regulated by various concentrations of NaCl over a 48-h period, and their expression levels under high-salt stress are relatively higher than those under either mild- or moderate-salt stress. In *R. trigyna*, the expression levels of *RtNHX1* in leaves showed an increase and reached a high level at 3 h, and then reduced after 6 h when exposed to high salt stress (200 mM NaCl) [22]. A similar expression pattern was found in sweet potato, where *IbNHX2* was significantly up-regulated at 4 h after treatment of 200 mM NaCl [51]. Lu et al. [60] found that the transcription level of *TaNHX3* in both leaves and roots sharply increased at 24 h and then gradually decreased after 48 h over a 96-h period in different wheat cultivars subjected to salt stress. Interestingly, more *TaNHX3* was detected in salt-tolerant cultivar “Ji7369” compared with salt-sensitive cultivars “Shimai12” and “Ji-Shi-3” [59]. Additionally, in *Porteresia coarctata*, the mRNA levels of *PcNHX1* in roots increased gradually up to 24 h and subsequently reduced to half of the initial level from 36 to 48 h after salt treatment and also upon salt withdrawal [61]. These results further confirmed that Vac-class NHXs play critical roles in the salt tolerance of plants. In this study, the expression levels of Vac-class *NHX* genes in leaves are significantly higher than those in roots under salt stress, implying that BvNHX1–3 can sequester Na^+^ in leaves during salinity stress. It was possible that, under mild-salt stress, Na^+^ accumulated in leaves of plants might be below vacuole capacity for compartmentalization Na^+^ [62], thus, in the present study the expression of *BvNHX1* and *-3* was relatively lower (Figure 6). Under high-salt stress, however, excessive Na^+^ was accumulated in leaves [59], here, *BvNHX1* and *BvNHX3* exhibited higher expression levels so that more Na^+^ could be compartmentalized in the vacuoles of leaves as soon as possible.

The plasma membrane Na^+^/H^+^ antiporters (PM-class NHX or SOS1) play important roles in extruding Na^+^ to the growth medium and/or controlling long-distance Na^+^ transport in plants [20,63,64,65]. In this study, *BvNHX5*/*BvSOS1* is significantly increased by salt stress. It is noticeable that its expression level is significantly higher in leaves than in roots under salt conditions, especially high-salt stress. In *Salicornia brachiate*, *SbSOS1* exhibited a greater level of constitutive expression in roots than in shoots and was further increased by salt stress [66]. Similar results were observed in *Puccinellia tenuiflora* [62] and *Z. xanthoxylum* [63]. These results proposed that BvSOS1 might be involved in response to salt stress. However, the precise mechanism of BvSOS1 in long-distance transport of Na^+^ needs to be addressed in future research.

### 4.4. The Protein-Protein Interaction Analysis Prediction

In this study, PPI analysis showed that NADH-cytochrome *b5* reductase 1 (CYR1, XP_010688037.1) is hypothesized interact with BvNHX1, -2, -3, and -4 (Figure 5). In all eukaryotes, CYR1 provides electrons, via cytochrome b5, for a range of biochemical reactions in cellular metabolism, including for fatty acid desaturation in the endoplasmic reticulum [67]. It is well documented that CYR1 has a crucial role in increasing the level of unsaturated fatty acids, which activates PM-H^+^-ATPase and, thus, reduces rhizosphere pH [68]. The results obtained from this study suggested that this protein might be involved in response to adversely environmental conditions.

Vac-class NHXs have been shown to be driven by electrochemical gradient of protons across tonoplasts generated by two vacuolar H^+^-pumps, H^+^-APTase and H^+^-PPase (VP) [9,10,69]. In this study, H^+^-PPase is hypothesized to interact with both BvNHX1 and -2, while H^+^-ATPase is hypothesized interact with BvNHX3 (Supplementary Figure S4). Co-expression of *ZxNHX* and *ZxVP1* genes significantly can significantly improve salt tolerance in transgenic plant species including *Lotus corniculatus* [70], alfalfa [23], and sugar beet [71], by increasing cation accumulation. These results implied that Vac-class NHXs might be cooperated with H^+^-PPase and H^+^-ATPase to transport Na^+^ across tonoplasts when plants were subjected to salt stress.

It is well-known that the calcineurin B-like (CBL) can interact and modulate the CBL-interacting protein kinases (CIPK), which, in turn, mediate Ca^2+^ signal transduction [33,72]. NHX7 (SOS1) is regulated by CBL and CIPK mediates the Ca^2+^ signaling pathway during salinity response [73]. In this pathway, a protein kinase complex consisting of CBL4 (SOS3) and CIPK24 (SOS2) was activated by a salt-stress elicited Ca^2+^ signal, and then the complex of CBL4-CIPK24 phosphorylated and activated the SOS1 protein to extrude Na^+^ out of cell in *Arabidopsis* under salt stress [74]. Overexpression of the *SOS1* gene also increased salt tolerance in transgenic tobacco by maintaining a higher K^+^/Na^+^ ratio [75]. In the current study, two CIPKs (CIPK8 and -24), and two CBLs are hypothesized to interact with BvNHX5 (Figure 5). Similarly, NHX7 (SOS1) interaction with CBLs and CIPKs were predicted in poplar [33] and *S. bicolor* [48]. Unlike BvNHX5, BvNHX1 is bound to the members of CIPK, but not to CBL (Supplementary Figure S4). These results suggested that BvNHX5 putatively interact with CBL and CIPK proteins different with other BvNHX proteins. However, these proteins interactions need to be further validated by yeast two hybrid in the future research.

## 5. Conclusions

In this study, we identified five putative *NHX* genes in the sugar beet genome. Phylogenetic analysis revealed that these *NHX* genes are grouped into three major classes, viz Vac-(*BvNHX1*, *-2* and *-3*), Endo- (*BvNHX4*), and PM-class *NHX* (*BvNHX5/BvSOS1*), and within each class the exon/intron structures are conserved. Amiloride-binding site (FFIYLLPPI) is found in TM3 at the N-terminus of BvNHX1, -2, and -3. *BvNHX5* contains one ABA responsive element, implying it might be involved in the ABA signal pathway. PPI network analysis revealed that only BvNHX5 putatively interacts with CBLs and CIPKs, suggesting this protein might be the primary NHX involved in CBL-CIPK pathway during salt stress response. Furthermore, qRT-PCR analysis indicated that all the *BvNHX* genes in leaves and roots are significantly up-regulated by salt, and their transcription levels under high-salt stress are relatively higher than those under low- and moderate-salt stress. These results suggested that the *BvNHX* genes play a vital role in sugar beet response to salt.

## Figures and Tables

**Table 1:** The sequences of primers used for qRT-PCR.

No.	Gene Name	Forward Primer Sequence (5’-3’)	Reverse Primer Sequence (5’-3’)
1	*BvACTIN*	ACTGGTATTGTGCTTGACTC	ATGAGATAATCAGTGAGATC
2	*BvNHX1*	TCGATGATTCTTTCATGAGG	GCCAACTGCCTCATACTCTG
3	*BvNHX2*	GTGTTAGATTTGGGTGTGCAAATAGC	GCAGTGATGGATTCATTGACCCAACG
4	*BvNHX3*	GTTTGGTGTACCAAATATAGTGCATG	TGATAGATTCATTAGCCCAACGATTC
5	*BvNHX4*	TCCTACAAATGATCCGCTGATATCTC	TGACGACGTAAAACATGACCAAGTAC
6	*BvNHX5*	GCCAGCTATGGCAGCTTATC	ATCCAAGGCCAATGCCGATG

**Table 2:** Identification of sugar beet *BvNHX* family genes.

Gene Name	Gene ID	Gene Code	Chr	Exons Count	CDS (bp)	ORF (aa)	pI	MW (kDa)	TM	Plant-mPLoc
*BvNHX1*	Xkmw	Bv1g006450_xkmw.t1	1	15	1659	552	6.31	61.3	12	Vac
*BvNHX2*	Zfce	Bv4g089680_zfce.t1	4	14	1560	519	8.45	58.2	11	Vac
*BvNHX3*	Jkji	Bv4g083400_jkji.t1	4	14	1617	538	6.33	59.3	12	Vac
*BvNHX4*	Pswr	Bv9g203100_pswr.t1	9	26	1584	527	5.5	58.2	12	Endo
*BvNHX5*	Sjdh	Bv6g131830_sjdh.t1	6	23	3489	1162	6.34	128.5	12	PM

Chr: chromosomal location; CDS: coding sequences; Endo: endosome; MW: molecular weight; ORF: open reading frame; PM: plasma membrane; pI: isoelectric point; TM: transmembrane domain; Vac: vacuole.

**Table 3:** Pairwise sequence similarity and divergence among BvNHX and AtNHX proteins.

		Similarity (%)											
	Protein names	BvNHX1	BvNHX2	BvNHX3	BvNHX4	BvNHX5	AtNHX1	AtNHX2	AtNHX3	AtNHX4	AtNHX6	AtHNX7	AtNHX8
**Divergence**	BvNHX1		78.8	79.6	64.4	8.6	89.2	89.5	78.5	84.0	65.0	10.1	43.1
	BvNHX2	57.7		77.8	65.5	9.1	79.5	79.5	86.5	79.1	65.8	11.1	43.3
	BvNHX3	61.3	68.9		65.0	9.4	79.9	79.5	79.1	78.2	64.9	11.1	43.0
	BvNHX4	183.2	166.1	184.7		10.1	65.2	64.4	64.9	64.7	86.8	12.0	43.6
	BvNHX5	299.0	257.0	265.0	237.0		9.4	9.1	9.5	8.9	9.8	61.9	43.8
	AtNHX1	25.2	57.5	62.6	177.6	265.0		94.2	79.5	83.7	65.4	10.6	43.3
	AtNHX2	25.3	56.5	63.5	186.7	281.0	12.3		79.4	84.1	65.5	10.6	43.2
	AtNHX3	62.4	35.2	62.9	178.0	252.0	59.3	60.3		78.7	65.6	11.1	43.8
	AtNHX4	35.4	59.9	61.9	170.1	264.0	37.6	36.5	62.5		66.4	10.3	42.7
	AtNHX6	175.9	175.9	191.7	30.9	244.0	174.1	172.2	177.3	168.4		11.8	43.3
	AtHNX7	274.0	229.0	243.0	221.0	50.8	252.0	255.0	236.0	248.0	226.0		51.0
	AtNHX8	267.0	245.0	273.0	254.0	46.8	254.0	261.0	241.0	255.0	261.0	31.1	

Similarity in upper triangle; divergence in lower triangle. Pair distance was calculated by Clustal W 2.0. Values marked with blue and grey colors represent divergence and similarity, respectively. The deeper background means the greater value. NHX: Na^+^/H^+^ antiporter.

**Table 4:** *Ks* and *Ka* substitution rates in *BvNHX* paralog genes.

Gene 1	Gene 2	*Ks*	*Ka*	*Ka*/*Ks*
*BvNHX1*	*BvNHX5*	0.1122	11.1040	99.0000
*BvNHX2*	*BvNHX5*	3.7373	7.9949	2.1392
*BvNHX3*	*BvNHX5*	18.8076	3.4894	0.1855
*BvNHX4*	*BvNHX5*	2.9900	5.5013	1.8399

*Ks*: synonymous substitution; *Ka*: non-synonymous substitution. *NHX*: Na^+^/H^+^ antiporter.

**Table 5:** Kinds and amounts of hormone-, stress-, and development-related *cis*-acting element in the promoters of *BvNHX* predicted by the PlantCARE tool.

Functional Class	Elements	Function	Sequence	Genes				
				*BvNHX1*	*BvNHX2*	*BvNHX3*	*BvNHX4*	*BvNHX5*
**Hormone**	ABRE	Abscisic acid-responsive element	ACGTG	0	0	0	0	1
	ERE	Ethylene-responsive element	ATTTCATA	1	0	1	1	0
	TCA-element	Involved in salicylic acid responsiveness	CCATCTTTTT	2	0	0	0	1
	TGA-element	Auxin-responsive element	AACGAC	0	0	1	0	1
**Stress**	ARE	Anaerobic-responsive element	AAACCA	1	0	0	2	0
	DRE	Drought-responsive element	GCCGAC	1	0	0	0	0
	LTR	Low-temperature responsiveness	CCGAAA	0	0	0	1	0
	WUN-motif	Wound-responsive element	AAATTACTA	0	0	1	0	0
	MYB	Drought-responsive element	CAACCA	1	0	0	0	0
	W box	Salt-responsive element	TTGACC	0	0	0	1	0
	STRE	Stress response element	AGGGG	0	0	0	0	1
**Others**	Box 4	Involved in light responsiveness	ATTAAT	1	0	1	0	0
	GT1-motif	Involved in light responsiveness	GGTTAAT	1	0	0	0	0
	TCT-motif	Involved in light responsiveness	TCTTAC	1	0	0	1	1
	Gap-box	Involved in light responsiveness	CAAATGAA	0	0	1	0	0
	AE-box	Modul for light response	AGAAACTT	0	0	0	1	0
	O2-site	Zein metabolism regulation	GATGACATGA	0	0	0	1	2
	G-box	Involved in light responsiveness	TACGTG	0	0	0	0	1

ABRE: Abscisic acid responsive element; ERE: Ethylene-responsive element; ARE: Anaerobic-responsive element; DRE: Drought-responsive element; MYB: Drought-responsive element; LTR: Low-temperature responsiveness; STRE: Stress response element.

**Table 6:** Structural dependent modeling parameters for the BvNHX proteins.

Protein	C-Score	TM-Score	RMSD (Å)	Best Identified Structural Analogs in PDB				
				PDB Hit	TM-Score ^a^	RMSD ^a^	IDEN ^a^	Cov
BvNHX1	−1.03	0.58 ± 0.14	9.9 ± 4.6	4cz9A	0.701	1.08	0.221	0.712
BvNHX2	−1.83	0.49 ± 0.15	11.8 ± 4.5	4cz9A	0.739	1.19	0.21	0.753
BvNHX3	−1.64	0.51 ± 0.15	11.4 ± 4.5	4cz8A	0.711	1.44	0.202	0.729
BvNHX4	−0.61	0.52 ± 0.15	11.3 ± 4.6	4cz9A	0.724	1.28	0.195	0.74
BvNHX5	−1.26	0.56 ± 0.15	12.4 ± 4.3	5ijoJ	0.902	1.59	0.087	0.915

C-score [−5, 2] is the confidence of each model, a higher value indicates a model with a higher confidence and vice-versa. TM-score and RMSD are determined based on the C-score value and the protein length following the correlation observed between these qualities. TM-score ^a^ indicates a measure of global similarity between query structure and known structure in PDB. RMSD ^a^ represents the RMSD between residues that are structurally aligned by TM-align. IDEN ^a^ is the percentage sequence identity in the structurally aligned region. Cov is the coverage of the alignment by TM-align and is equal to the number of structurally aligned residues divided by length of the query protein.

## Supplementary Materials

The following are available online at https://www.mdpi.com/2073-4425/10/5/401/s1, **Table S1.** The genomic DNA sequences, mRNA sequences, CDS, protein sequences, phosphorylation sites, and phylogeny sequences. **Figure S1.** Transmembrane domains for BvNHX proteins. **Figure S2.** Details of motifs in BvNHX proteins identified by MEME. **Figure S3.** Details of secondary structure of BvNHX proteins. **Figure S4.** String analysis for individual BvNHX protein.

## Abbreviations

ABA	Abscisic acid
ABRE	ABA-responsive element
ARE	Anaerobic-responsive element
ATM	Ataxia telangiectasia mutated
CBL	Calcineurin B-like proteins
CDC2	Cell division cycle protein 2
CDS	Coding sequences
CIPK	CBL-interacting protein kinases
CMP	Calcium-binding protein
CYB5R1	NADH-cytochrome *b*5 reductase 1
DER	Drought-responsive element
ERE	Ethylene-responsive element
GSDS	Gene structure display serve
GSK3	Glycogen synthase kinase 3
HKT	High-affinity K^+^ transporter
IAA	Auxin
KEA	K^+^ efflux antiporter
LTR	Low-temperature responsiveness
ORF	Open reading frame
PDB	Protein data bank
pI	Isoelectric point
PKA	Protein kinase A
PKC	Protein kinase C
PM	Plasma membrane
PPI	Protein-protein interaction
SA	Salicylic acid
SOS1	Salt overly sensitive 1
SERE	Stress response element
3-D	Three-dimension
TM	Transmembrane helical domain
VP	Vacuolar H^+^-PPase
MEME	Multiple expectation maximization for motif elicitation
MW	Molecular weight
NHX	Na^+^/H^+^ antiporter
