# Supplementary figures and images for: Genome-Wide Identification of Na+/H+ Antiporter (NHX) Genes in Sugar Beet (Beta vulgaris L.) and Their Regulated Expression under Salt Stress
Source: Genes (Basel). 2019 May 27;10(5):401. doi: 10.3390/genes10050401 (PMC6562666; doi:10.3390/genes10050401)

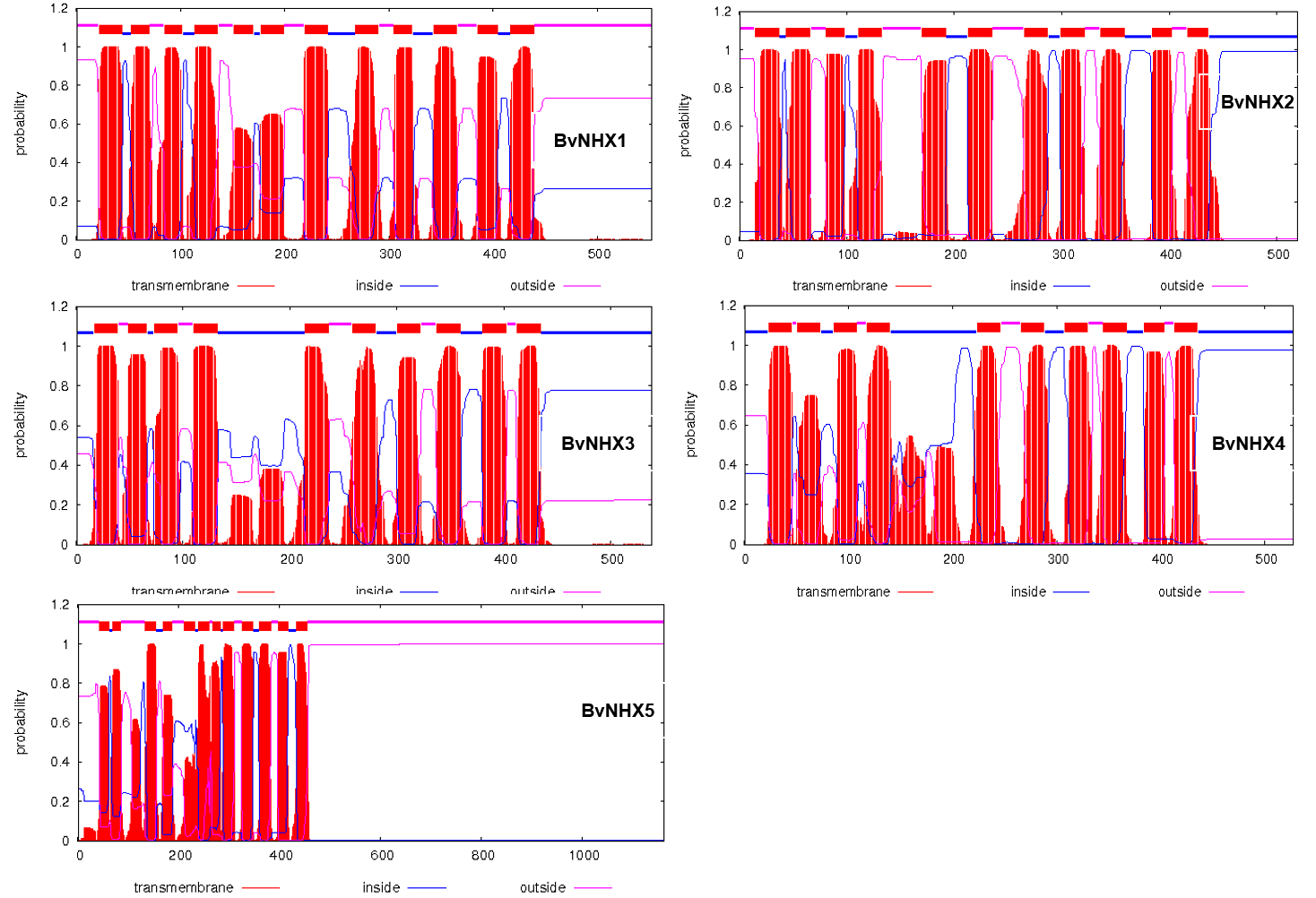

Supplement: Supplementary file 1 [file genes-10-00401-s001.zip › SUPPLEMENTARY MATERIALS/Figure S1.png]

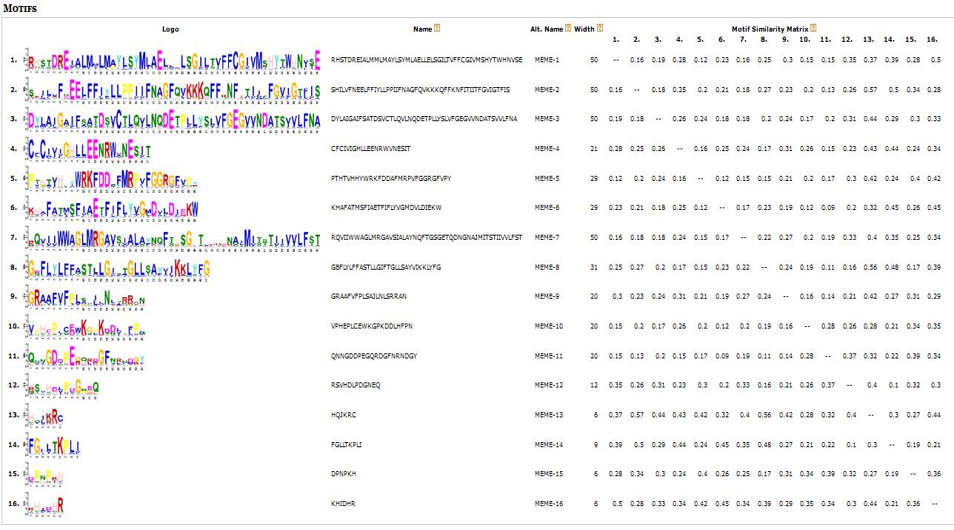

Supplement: Supplementary file 1 [file genes-10-00401-s001.zip › SUPPLEMENTARY MATERIALS/Figure S2.png]

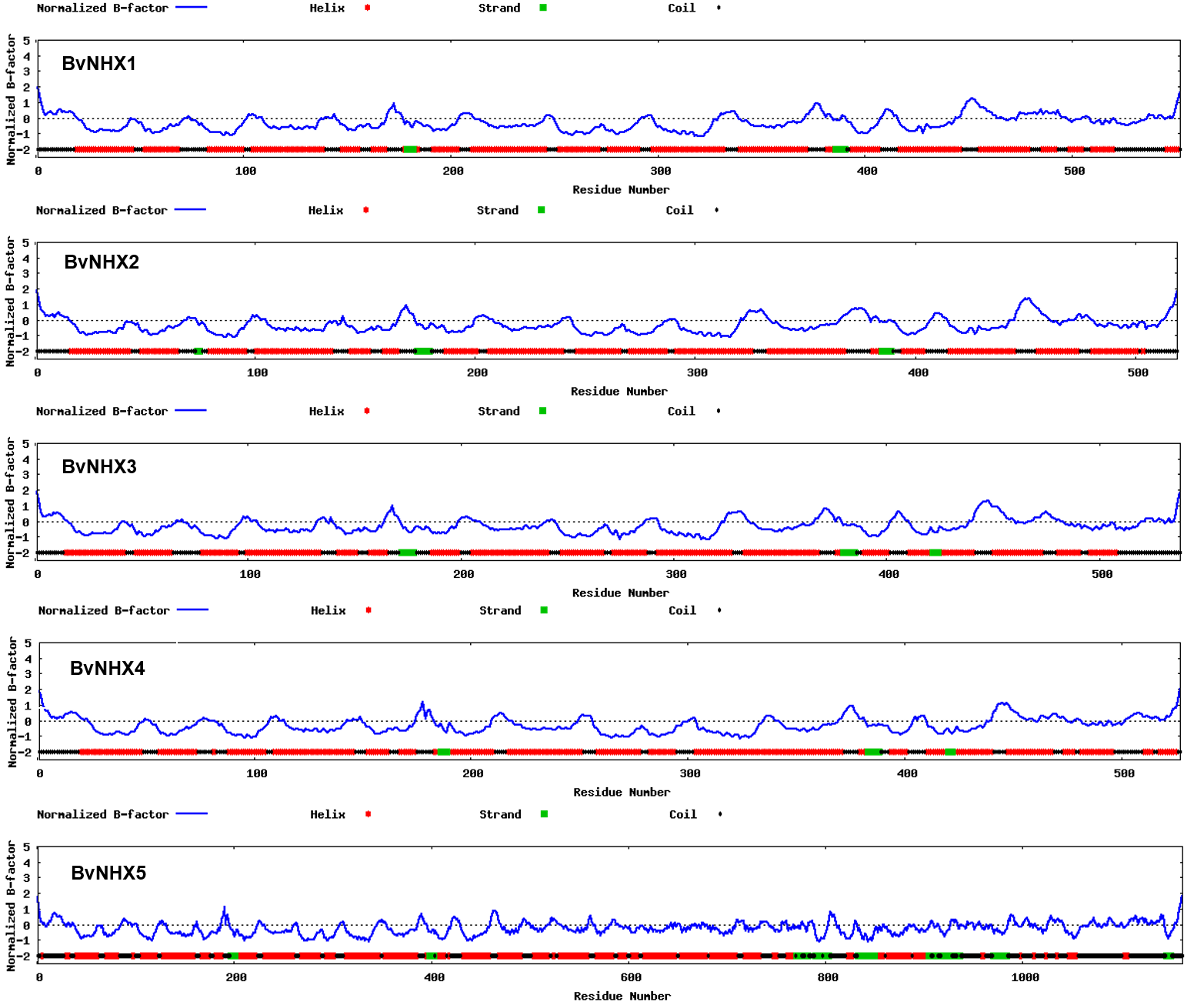

Supplement: Supplementary file 1 [file genes-10-00401-s001.zip › SUPPLEMENTARY MATERIALS/Figure S3.png]

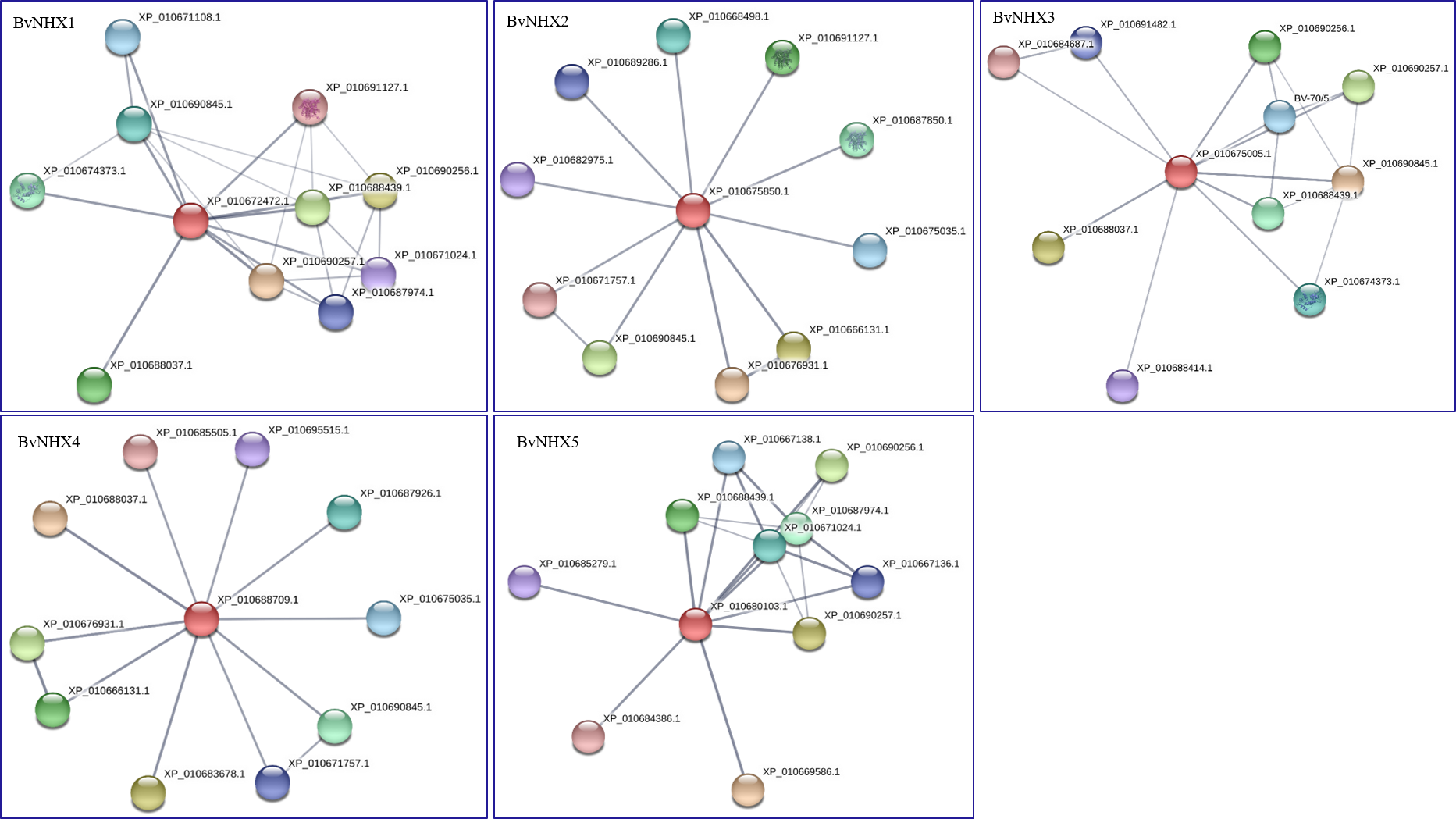

Supplement: Supplementary file 1 [file genes-10-00401-s001.zip › SUPPLEMENTARY MATERIALS/Figure S4.png]
